# Unveiling the *Petunia hybrida* Virome: Metatranscriptomic Profiling from the Bulgarian Market and In Vitro Cultures

**DOI:** 10.3390/plants14162597

**Published:** 2025-08-21

**Authors:** Rumyana Valkova, Stoyanka Jurak, Elena Apostolova-Kuzova, Vesselin Baev, Lilyana Nacheva, Galina Yahubyan, Dijana Škorić, Mariyana Gozmanova

**Affiliations:** 1 Department of Molecular Biology 24, University of Plovdiv, Tsar Assen Str., 4000 Plovdiv, Bulgaria; boncheva@uni-plovdiv.bg (R.V.); tanyajurak@uni-plovdiv.bg (S.J.); eapostolova@uni-plovdiv.bg (E.A.-K.); baev@uni-plovdiv.bg (V.B.); gyahubyan@uni-plovdiv.bg (G.Y.); 2 Fruit Growing Institute, Agricultural Academy, 12 Ostromila Str., 4004 Plovdiv, Bulgaria; lilyn@abv.bg; 3 Department of Biology, Faculty of Science, University of Zagreb, Marulićev trg 9a, 10000 Zagreb, Croatia

**Keywords:** petunia, HTS/Illumina, PVCV, CMV, TAV, RNA sequencing, in vitro collection

## Abstract

RNA sequencing is a high-throughput sequencing method essential for unbiased detection and characterization of known and emerging plant viruses. Its high sensitivity makes it particularly well-suited for identifying low-abundance viral sequences, even in asymptomatic plants or those affected by complex, mixed infections. Here, we conducted a metatranscriptomic survey of *Petunia hybrida* plants from the Bulgarian market, both symptomatic and asymptomatic, and their corresponding in vitro plantlets. Viruses were detected in all tested samples demonstrating that visual symptoms are not a reliable indicator of infection. The viromes were dominated by petunia vein clearing virus (PVCV, *Petuvirus venapetuniae*), cucumber mosaic virus (CMV, *Cucumovirus CMV*), and tomato aspermy virus (TAV, *Cucumovirus TAV*), along with bacteriophages and fungus-associated viruses. However, the PVCV and CMV abundance was elevated in in vitro samples, possibly due to cutting-induced activation and/or prolonged cultivation. Phylogenetic analysis of the Bulgarian CMV, TAV, and PVCV isolates highlights their genetic links to strains from a wide geographic range and diverse hosts, emphasizing the potential for virus movement and genetic exchange among plant viruses across regions and species. It also suggests that petunias may contribute to the transmission dynamics of viruses within ornamental trade networks. These findings also emphasize the phytosanitary risks to horticulture and establish a basis for further investigation into plant virus ecology.

## 1. Introduction

The genus *Petunia*, a member of the Solanaceae family, comprises herbaceous perennial plants that are cultivated as annuals for ornamental purposes. *Petunia hybrida*, the most widely grown species, is a complex hybrid derived primarily from *P. axillaris* and species or subspecies of *P. integrifolia* [1,2]. This hybridization, followed by nearly two centuries of intensive breeding, has resulted in a broad range of cultivars exhibiting remarkable variation in flower color, shape, size, and growth habit [3,4,5,6]. Petunias hold significant commercial value in global horticulture with an estimated market worth of $141.7 million in the United States [7]. The global economic importance of petunias is further supported by high seed trade.

Cultivating petunias across varying environmental conditions elevates the risk of infection by a broad array of pathogens. The viral pathogens comprise members of the *Alfamovirus*, *Cucumovirus*, *Potyvirus*, *Tobamovirus*, *Orthotospovirus*, *Fabavirus*, and *Nepovirus* genera, together with viroid representatives of the *Pospiviroidae* family, which are transmitted through mechanical means, insect vectors (aphids, thrips, nematodes), or contaminated seeds [8,9,10,11]. Tomato mosaic virus (ToMV, *Tobamovirus tomatotessellati*) and tobacco mosaic virus (TMV, *Tobamovirus tabaci*) are predominantly mechanically transmitted, while petunia vein clearing virus (PVCV, *Petuvirus venapetuniae*), a member of the *Petuvirus* genus in the family *Caulimoviridae*, is vertically transmitted through the host genome. Notably, cultivated petunias can harbor more than 50 PVCV proviral copies integrated into their genome, likely originating from wild species such as *P. axillaris* [12].

Viruses are important constituents of the phytobiome, yet our understanding of their interactions with host plants remains limited. Mixed viral infections within individual plants are now recognized as common occurrences, with the potential to influence disease progression, host responses, and the persistence of pathogens [13]. Despite these insights, substantial knowledge gaps persist regarding virome composition, ecological dynamics, and their overall impact on plant health. Plant tissue culture provides a reliable platform for in vitro studies of virus–host interactions by preserving viral and microbial communities, especially when larger stem explants are used [14]. Additionally, it enables the propagation of genetically uniform plantlets under controlled conditions, ensuring reproducibility in viral pathogenesis research. The utility of in vitro systems in virology was first demonstrated by White (1934), who cultured excised tomato roots infected with TMV [15]. Subsequent studies confirmed the capacity of callus and cell suspension cultures of tomato (*Solanum lycopersicum*) and potato (*S. tuberosum*) to support persistent viroid replication [16,17,18,19]. These systems have become valuable models for studying viral replication, pathogenesis, and host responses under defined conditions. For virus detection in both greenhouse-grown and tissue-cultured plants, several diagnostic tools such as electron microscopy, serological assays, and nucleic acid-based techniques have been applied. Nevertheless, these methods may fail to detect viruses present at low concentrations or those that are previously uncharacterized. In such cases, high-throughput sequencing (HTS) provides a powerful, unbiased alternative capable of identifying a broad spectrum of viral pathogens without prior sequence information [20,21,22,23,24].

In Bulgaria, petunias are extensively cultivated for ornamental landscaping and garden decoration. Recognizing the growing commercial and ecological significance of ornamental plant viromes, we conducted a metatranscriptomic survey of *P. hybrida* specimens sourced from commercial markets in Bulgaria, alongside their in vitro-propagated counterparts. Our objectives were to characterize the composition, diversity, and dynamics of petunia-associated viruses, and to evaluate the potential of in vitro systems for virome studies. The findings offer valuable insights into virus–host coexistence, highlight the role of asymptomatic infections in virus transmission, and underscore the phytosanitary risks associated with the petunia trade.

## 2. Results

### 2.1. Phenotypic and Photosynthetic Alterations in Symptomatic and Asymptomatic Petunia Plants

**Figure 1:**
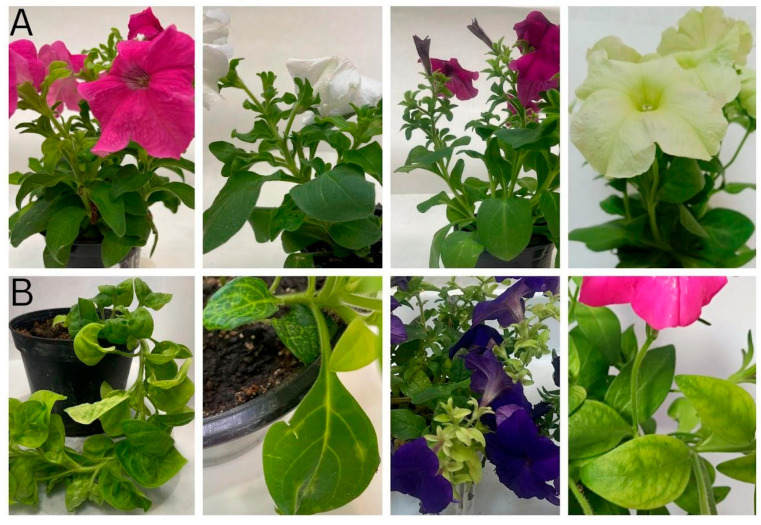
The phenotype of selected asymptomatic (**A**) and symptomatic (**B**) petunias collected from the Bulgarian market.

*P. hybrida* plants were collected from the market and categorized into two groups. The first group included ten visually healthy (asymptomatic) plants with no phenotype changes (Figure 1A), while the second consisted of seventeen plants displaying symptoms such as chlorosis, leaf deformation, curling, and vein clearing (Figure 1B), suggesting possible viral infection.

**Figure 2:**
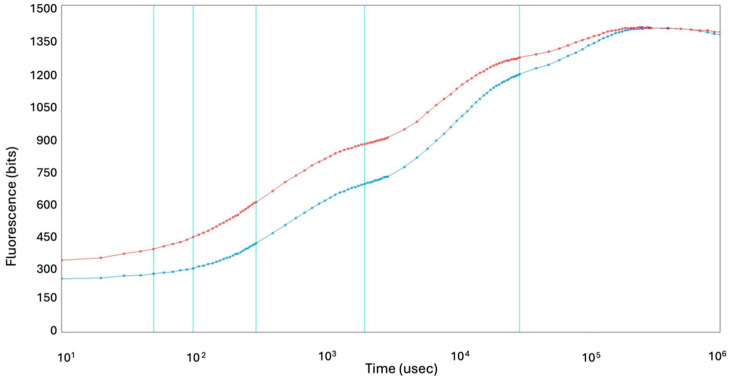
OJIP chlorophyll a fluorescence induction curves. Blue line—asymptomatic market collected plants, red line—symptomatic market plants.

The plant health was assessed by employing the chlorophyll *a* fluorescence induction parameters (OJIP), which enable early detection of stress-induced photosynthetic disturbances. The symptomatic plants exhibited significantly higher baseline fluorescence (F_0_ = 334 ± 25) compared to asymptomatic ones (F_0_ = 241 ± 7), indicating impaired excitation energy transfer at the PSII reaction centers (Figure 2, Table S1). Furthermore, a reduction in the maximum quantum yield of PSII photochemistry (Fv/Fm) was observed in the symptomatic plants (0.761 ± 0.006) relative to the asymptomatic ones (0.828 ± 0.006), suggesting photoinhibition or structural damage to PSII. Other OJIP-derived parameters reported in Table S1 also differed between the groups, further underscoring the physiological stress in symptomatic plants. However, the potential presence of viruses and their delayed effects on asymptomatic individuals warrant further investigation.

### 2.2. In Vitro Culture of Symptomatic and Asymptomatic Plantlets

**Figure 3:**
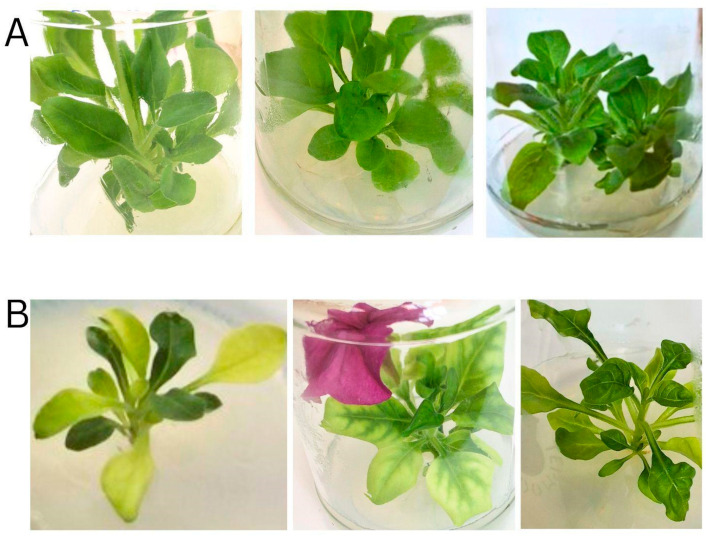
In vitro-derived *P. hybrida* plantlets: (**A**) Asymptomatic; (**B**) Symptomatic plants.

In vitro cultures, maintained under aseptic conditions to eliminate the introduction of new pathogens and reduce the influence of abiotic stress through environmental control, provide a stable system for virome analysis. Axillary buds from both symptomatic and asymptomatic plants were surface-sterilized and cultured under aseptic conditions to establish in vitro plantlets (Figure 3). The in vitro plantlets showing virus-like symptoms resembling their parent plants were selected for further analysis.

### 2.3. Virus Detection by Long Non-Coding RNA Sequencing

Leaves from six asymptomatic and six symptomatic plants from both market and in vitro sources were pooled into four RNA groups: Mas (market asymptomatic), Ms (market symptomatic), Ias (in vitro asymptomatic), and Is (in vitro symptomatic), and subjected to long non-coding RNA sequencing (lncRNA sequencing). We used this sequencing approach because its sensitivity in detecting low-abundance viral sequences makes it particularly valuable for analyzing asymptomatic plants or plants affected by complex, mixed infections. Market samples yielded 47,025,103 (Mas) and 48,897,157 (Ms) raw reads (Table S2). Viral reads represented 0.00531% in Mas and were nearly 8-fold higher in Ms (0.0402%). In vitro samples produced 46,380,965 (Ias) and 48,016,732 (Is) reads, with viral reads comprising 0.171% and 0.155%, respectively. In vitro plantlets demonstrated increased viral abundance compared to market plants, most notably in Ias, where viral reads were approximately 32 times higher than in Mas.

### 2.4. Taxonomic Composition of the Petunia Virome Across Market and In Vitro Samples

**Figure 4:**
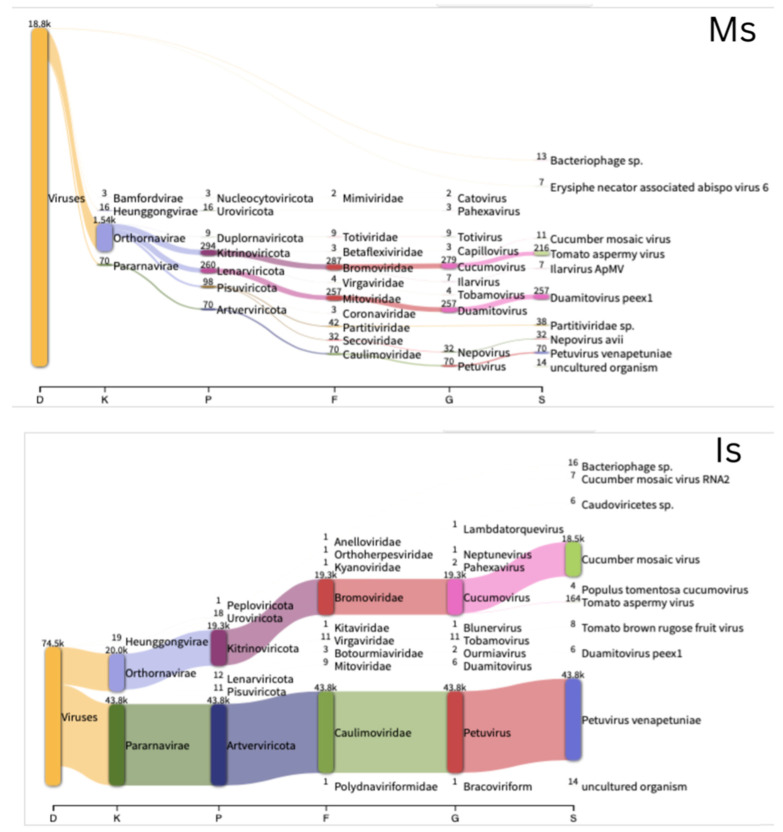

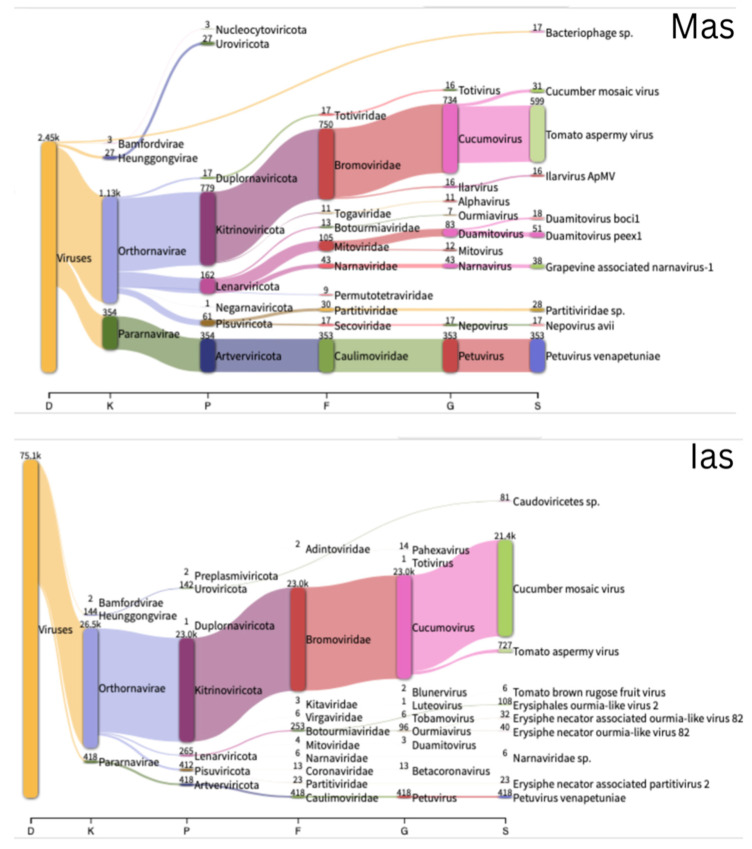
Classification of viral sequences detected in petunia samples illustrated by Sankey diagrams. The classification follows hierarchical taxonomic levels from kingdom to species. Mas = asymptomatic market; Ms = symptomatic market; Ias = asymptomatic in vitro; Is = symptomatic in vitro. In the figure, the virus names were generated automatically by the program according to the Kraken database, so some viruses are represented erroneously by their non-italicized species names; petunia vein clearing virus (PVCV) is represented as Petuvirus venapetuniae.

Taxonomic analysis from both market and in vitro samples consistently revealed the presence of *Caulimoviridae* and *Bromoviridae* at the family level (Figure 4, Table S3). In Ms, *Bromoviridae* accounted for 40.4% of the viral community, followed by *Mitoviridae* at 36.2% and *Caulimoviridae* at 9.9%. In Mas, *Bromoviridae* remained dominant (54.2%), while *Caulimoviridae* and *Mitoviridae* represented 25.5% and 7.5%, respectively. In Is, *Caulimoviridae* was more prevalent, comprising 69.4% of the viral population, while *Bromoviridae* constituted 30.5%. In Ias, *Bromoviridae* dominated the virome, making up 97% of the viral content, while *Caulimoviridae* was present at 1.8%. Despite variations in relative abundance across samples, certain viral genera—*Petuvirus* and *Cucumovirus*—were consistently detected, indicating a stable association with the petunias studied.

At the species level, the Ms sample harbored a substantial proportion (37%) of Petunia exserta mitovirus 1 (PeexMV1, *Duamitovirus peex1*), typically associated with the mitochondria of plant-pathogenic fungi, along with tomato aspermy virus (TAV, *Cucumovirus TAV*) (36.9%) and PVCV (10%) (Figure 4). In Mas, TAV accounted for 45.5%, followed by PVCV at 27%. PeexMV1 was also detected at a relatively low level (3.9%). In Is, PVCV (70.1%) and cucumber mosaic virus (CMV, *Cucumovirus CMV*) (29.6%) were the dominant species, while TAV was detected at a much lower abundance (0.3%). In Ias, CMV was the most prevalent species, representing 93.5% of the viral reads. TAV (3.2%) and PVCV (1.8%) were also identified. Additionally, erysiphales ourmia-like virus 2 (0.5%) was detected, which may indicate potential plant–fungus interactions.

Since RNA sequencing was performed on pooled samples, PCR or RT-PCR was applied to confirm the presence of the predominant viruses—PVCV, TAV, and CMV in individual plants (Table 1).

The results show that CMV and PVCV were present in petunia samples, regardless of the symptom expression or propagation method. In certain individuals (43, 47 and 31) the two viruses were found only in the in vitro counterparts. This widespread occurrence suggests that CMV and PVCV are persistent even in absence of symptoms in the studied petunias indicating a certain level of tolerance in these plants. TAV was only detected in some of the in vitro and in vivo plants analyzed (41, 42, 48, 33 and 35), suggesting a lower prevalence.

**Figure 5:**
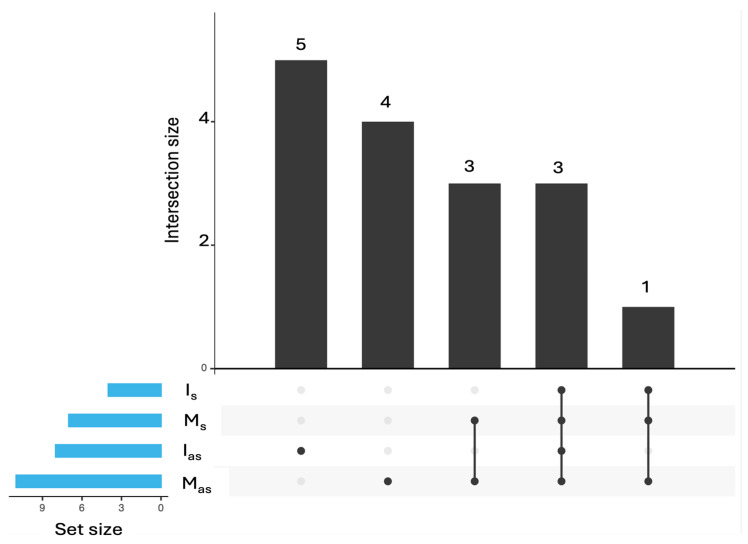
UpSet plot showing shared and unique viruses across symptomatic and asymptomatic petunia samples from different sources. M_as_ = asymptomatic market; M_s_ = symptomatic market; I_as_ = asymptomatic in vitro; I_s_ = symptomatic in vitro.

The common and unique viral species identified in the analyzed petunia samples, as revealed by the UpSet diagram (Figure 5) and Table 2.

Three viruses—CMV, TAV, and PVCV—were consistently detected across all sample types, including both symptomatic and asymptomatic plants from market and in vitro sources, highlighting their widespread presence. Beyond these common viruses, species from the families *Mitoviridae* (such as *Duamitovirus peex 1*), *Partitiviridae*, and *Secoviridae* (such as *Nopovirus avii*), indicating the presence of Petunia exserta mitovirus 1 and cherry leafroll virus, respectively, were shared between symptomatic and asymptomatic market plants, suggesting a common viral background in these groups. The asymptomatic market plants also harbored a small group of unique viruses, including apple mosaic virus (sequenes belonging to the species *Ilarvirus ApMV*), Botrytis cinerea alpha-like virus 1 (*Betasclernavirus botrytidis*), Botrytis cinereal mitovirus 1 (*Duamitovirus boci1*), and grapevine associated narnavirus-1, indicating a more diverse virome composition. Interestingly, the virome of symptomatic in vitro plants included *Bacteriophage* sp. shared with market-sourced groups. This suggests that in vitro culture conditions tend to reflect the virome composition of market plants, regardless of symptom development. Regarding asymptomatic in vitro plants, viral sequences corresponding to Erysiphe-associated viruses (likely mycoviruses infecting the fungal pathogen *Erysiphe necator*) and *Caudoviricetes* sp. members (bacteriophages) were detected, despite the absence of visible fungal or bacterial contamination. Their presence may be attributed to the high sensitivity of metatranscriptomic sequencing, which enables the detection of low-abundance viral sequences even in the absence of overt infection.

### 2.5. Phylogenetic Analysis of Plant Viruses in Bulgarian Petunia Isolates

In our study, we detected TAV, CMV, and PVCV co-infecting petunia plants in Bulgaria, providing further evidence of complex viral interactions (Table 1).

**Figure 6:**
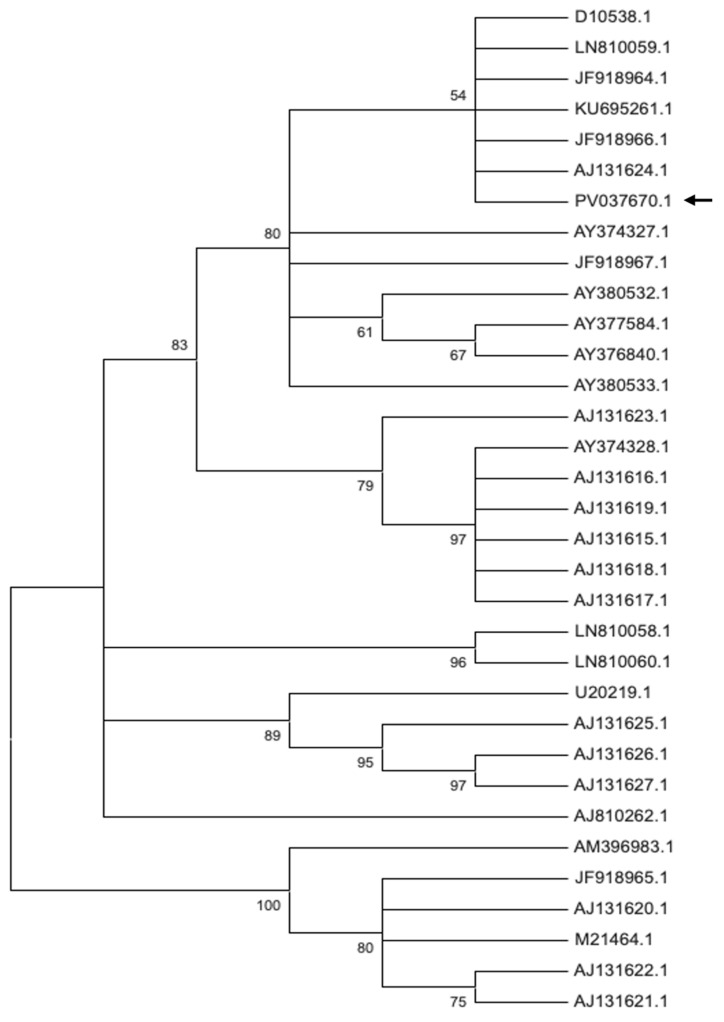
The phylogeny of Bulgarian cucumber mosaic virus isolates from *P. hybrida.* The phylogenetic tree was constructed using the Maximum Likelihood method and Tamura–Nei model, conducted in MEGA 12. The analytical procedure encompassed 33 nucleotide sequences with 428 positions of the CP gene in the final dataset. The percentage of replicate trees in which the associated taxa clustered together (1000 replicates) is shown above the branches. The arrow indicates the analyzed Bulgarian accession available in NCBI GenBank.

For CMV, we analyzed the coat protein (CP) gene sequence of the Bulgarian isolate (GenBank accession no. PV037061.1) in comparison with reference strains representing subgroups IA, IB, and II, as well as with CMV sequences from ornamental plants and additional Bulgarian accessions available in NCBI GenBank (Figure 6, Table S4a). Phylogenetic analysis revealed that isolate PV037061.1 clusters within CMV subgroup IA. Notably, it exhibits high genetic similarity to LN810059.1 (isolated from *Citrullus lanatus* in Greece, a region geographically proximate to Bulgaria), as well as to JF918966.1 and JF918964.1 (both from *Vinca minor* in the USA), and KU69526.1 (from *Dimorphotheca caulescens* in Iran).

**Figure 7:**
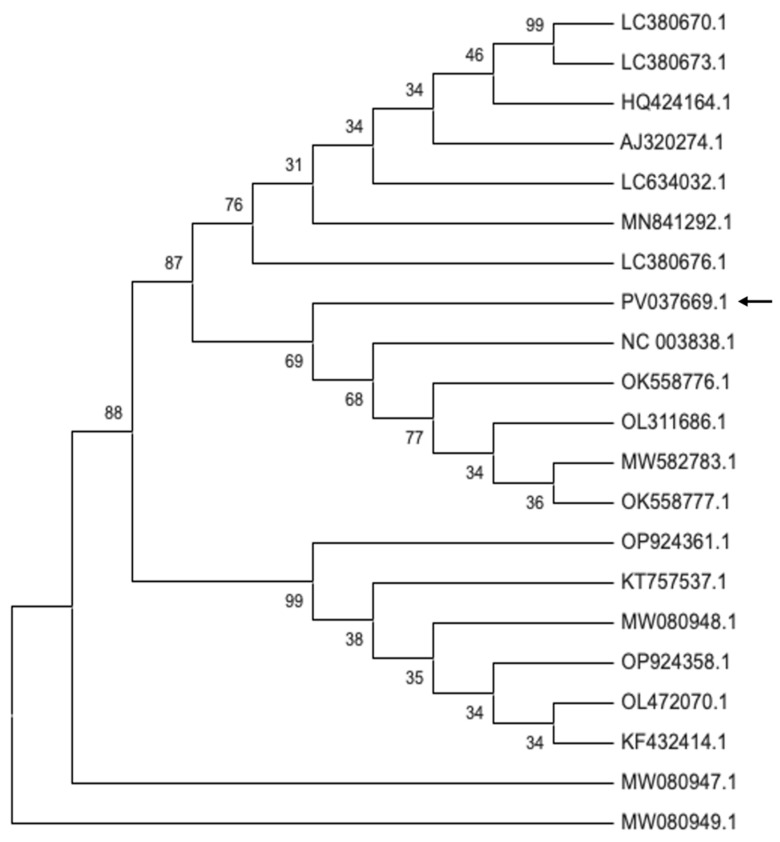
Molecular phylogenetic analysis of Bulgarian tomato aspermy virus isolates from *P. hybrida.* The phylogenetic tree was constructed using Maximum Likelihood method and Tamura–Nei model based on 21 nucleotide sequences with 947 positions of the RdRp gene in the final dataset. Evolutionary analysis was conducted in MEGA12. The percentage of replicate trees in which the associated taxa clustered together (1000 replicates) is shown above the branches. The arrow indicates the analyzed Bulgarian accession available in NCBI GenBank.

The RNA-dependent RNA polymerase (RdRp) sequence of the Bulgarian TAV isolate PV037669.1 was compared with RdRp sequences from TAV isolates infecting various Solanaceae hosts (Figure 7, Table S4b). Phylogenetic analysis showed that the petunia isolate PV037669.1 clustered within a subgroup that includes an isolate from Spain (NC_003838.1), two isolates from Canada (OK558776.1, OK558777.1), and two TAV isolates from Germany—one from chrysanthemum (OL311686.1) and one from tomato (MW582783.1).

**Figure 8:**
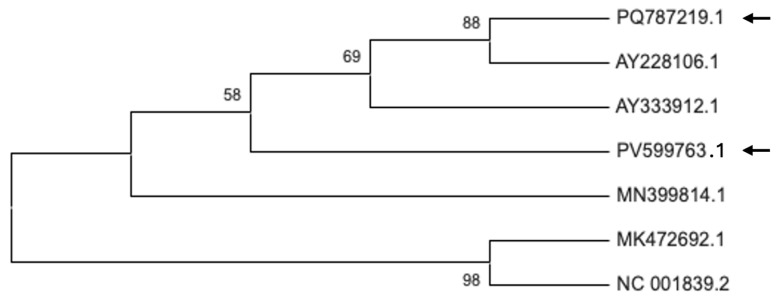
The phylogeny of Bulgarian petunia vein clearing virus isolates from *P. hybrida*. The phylogenetic tree was constructed using Maximum Likelihood method and Tamura–Nei model based on 7 nucleotide sequences with 654 positions of the polyprotein gene in the final dataset. Evolutionary analyses were conducted in MEGA12. The percentage of replicate trees in which the associated taxa clustered together (1000 replicates) is shown next to the branches. The arrows indicate the analyzed Bulgarian accessions available in NCBI GenBank.

The phylogenetic relationships of the Bulgarian PVCV isolates PV599763.1 and PQ787219.1 were inferred based on the reverse transcriptase domain of the polyprotein sequence (Figure 8, Table S4c). These two isolates clustered with AY228106.1, AY333912.1 (The Netherlands), and MN399814.1 (South Korea), suggesting a shared evolutionary origin.

## 3. Discussion

Petunia plants are susceptible to a range of pathogens, including bacteria, viruses, fungi, and phytoplasmas; nonetheless, viral pathogens affecting the Bulgarian petunias market remain insufficiently investigated. This study aimed to characterize the virome composition, prevalence and genetic diversity of viruses affecting *P. hybrida*. Different factors, such as virus strain, environmental conditions, host genotype, and mixed infections, can influence the phenotypic viral appearance, and even some infected plants may remain asymptomatic [8,25]. To objectively evaluate plant physiological status, chlorophyll a fluorescence (OJIP test) was used [26]. Symptomatic plants exhibited increased baseline fluorescence (F_0_) and reduced maximum quantum efficiency (Fv/Fm), confirming photosynthetic impairment (Figure 2, Table S1). These findings confirm physiological disturbances in the symptomatic plants, while potential latent effects in asymptomatic ones require further investigation. Since visible phenotypic traits alone do not always reliably distinguish healthy from diseased plants, chlorophyll fluorescence provided an objective parameter to assess the physiological state and detect early disruptions in the photosynthetic apparatus.

RNA sequencing, combined with PCR/RT-PCR and Sanger sequencing, enabled the molecular detection of viruses present in the studied petunias. Three prevalent viruses were detected: two RNA viruses from the *Bromoviridae* family (CMV and TAV) and one DNA virus from the *Caulimoviridae* family (PVCV). Most market plants were co-infected with multiple viruses, which emphasized complex virus-virus and virus–host interactions (Table 1). Similar mixed infections involving CMV and TAV, in combination with other viruses, have previously been documented in the ornamental plant *Hippeastrum hybridum* in Bulgaria [27]. Detection of the three viruses (TAV, CMV and PVCV) in the asymptomatic petunias highlights the importance of molecular diagnostics for effective phytosanitary management, as the phenotype alone does not reliably indicate the presence of viral infections. These viruses may persist latently and maintain stable associations with the host regardless of external stressors or phenotypic expression. To our knowledge, the current study is the first on the petunia virome from the Bulgarian market.

In vitro cultivation was reported to propagate virus infection and affect the virome distribution [14,22,28], such changes can be characterized in greater detail using NGS [22]. In our study, we established in vitro cultures from both symptomatic and asymptomatic market-sourced petunia plants and applied NGS to assess changes in virome composition. We found that both CMV and PVCV were more abundant in the in vitro samples compared to the original market plants (Figure 4). This result is not unexpected, as tissue culture procedures involve repeated cutting, a process known to induce DNA demethylation at specific genomic loci—an effect previously linked to the activation of endogenous pararetroviral sequences [12,29]. Recent findings indicate that the infectious status and genetic diversity of grapevine viruses remain stable in long shoot tip cultures even after four subcultures. Notably, in vitro grapevine plants exhibited a much higher viral titer compared to their in vivo counterparts [14]. Conversely, PeexMV1 was absent in in vitro samples, suggesting possible elimination during surface sterilization for culture initiation. The detection of non-plant viruses, such as fungi- and bacteriophage-associated sequences, in asymptomatic in vitro plants highlights the complexity of plant–microbe interactions under sterile conditions. These results emphasize the need for integrated molecular screening in the established in vitro model system, as low-abundance viruses can persist without visible symptoms or overt contamination.

The phylogenetic analysis of the Bulgarian CMV isolate PV037670.1, shows that it belongs to subgroup IA. This isolate demonstrates close genetic similarity to strains from Greece, as well as to isolates from ornamental plants from the USA, the Netherlands, and Iran, indicating a broad genetic similarity among subgroup IA members across different regions and host species. The Bulgarian TAV isolate PV037669.1, clusters with isolates from Spain, Canada, and Germany, reflecting close genetic ties to TAV strains from both neighboring and distant countries, as well as from various hosts within the Solanaceae family and related ornamentals. The Bulgarian PVCV isolates PV599763.1 and PQ787219.1, group with isolates from the Netherlands and South Korea, suggesting genetic connectivity across Europe and Asia. Collectively, these findings highlight that the Bulgarian CMV, TAV, and PVCV isolates are genetically linked to strains from a wide geographic range and diverse hosts, emphasizing the potential for virus movement and genetic exchange among plant viruses across regions and species.

## 4. Materials and Methods

### 4.1. Plant Sampling

A total of 27 *P. hybrida* plants, both symptomatic and asymptomatic, were collected during the blooming stage from commercial nurseries in the Plovdiv region, South-East Bulgaria (42.1354° N, 24.7453° E) between 2023 and 2024. Plants were transferred to the laboratory and maintained under controlled conditions (16/8 h light/dark photoperiod at 24 °C) for one month. Two to three leaves were harvested per plant and rinsed with distilled water (ddH_2_O) to remove dust and epiphytic organisms. These samples were subsequently used for RNA extraction.

### 4.2. Chlorophyll a Fluorescence Assay and Chlorophyll Content

Chlorophyll *a* fluorescence induction (OJIP) curves were measured on the first fully expanded leaf of five studied plants of each group using a Plant Efficiency Analyzer (Handy-PEA, Hansatech Instruments Ltd., Norfolk, UK). Before measurement, leaf areas were dark-adapted for 40 min using leaf clips. Measurements were taken under 1 s of illumination at 3000 µmol m^−2^ s^−1^ PPFD. Data were processed using PEA Plus Software (v1.10) and interpreted following the protocols of Strasser and Strasser [30] and Goltsev and co-authors [31].

The chlorophyll content was measured using the portable chlorophyll meter CL-01 (Handy-PEA, Hansatech Instruments Ltd., Norfolk, UK) on the leaf used for chlorophyll fluorescence assessment.

### 4.3. Establishment of In Vitro Cultures from Axillary Buds

Young stem segments without flowers were rinsed under running tap water to remove surface debris. The large leaves were removed before sterilization, while smaller leaves were excised post-sterilization. Surface sterilization was carried out using 70% ethanol for 10 s, followed by 1% sodium hypochlorite for 30 min. Segments were then rinsed three times with sterile distilled water, dried on sterile filter paper, and cut into 15 mm explants. Explants were placed in test tubes containing sterile half-strength Murashige and Skoog (1/2 MS) medium solidified with agar [32]. Cultures were maintained at 24 ± 1 °C under a 16/8 h light/dark cycle. After four weeks, sterile plantlets were transferred to full-strength MS medium in larger vessels and subcultured every four weeks.

### 4.4. Long Non-Coding RNA Sequencing and Bioinformatics Analysis

Total RNA was extracted from 0.1 g of leaf tissue using the innuPREP Plant RNA Kit (Analytik Jena, Jena, Germany). RNA concentration and integrity were assessed using the Qubit 4 Fluorometer. Equimolar RNA pools were prepared from symptomatic and asymptomatic plants for sequencing. Ribosomal RNA was depleted, and libraries were prepared by Novogene Ltd., Cambridge, UK. Sequencing was performed on a NovaSeq X Plus Series (PE150) platform. Raw reads were deposited in the SRA database (accession number PRJNA1276299). Quality trimming was performed using TrimGalore (v6.7). Taxonomic classification was executed with Kraken2 (v2.1.3) based on raw read counts and employing the core_nt Database 28 December 2024. Classification outputs were visualized with Pavian (v1.0) to assess taxonomic composition. UpSet analysis was conducted using the E Venn web tool [33,34] to visualize intersections of viral taxa across sample groups. A read count threshold of >10 was applied.

### 4.5. PCR and RT-PCR Virus Amplification and Phylogenetic Analysis

CMV, TAV and PVCV were detected using a one-step cDNA RT-PCR kit (Genaxxon Bioscience GmbH, Ulm, Germany). Virus-specific primers are listed in Table S5. For Sanger sequencing: first-strand cDNA was synthesized from 500 ng of total RNA using the SCRIPT cDNA synthesis kit (Jena Bioscience, Jena, Germany) with random hexamer primers. PCR amplification was performed with Pfu X polymerase (Jena Bioscience GmbH, Jena, Germany) following the manufacturer’s protocol (Table S6). PCR products were purified from agarose gels using the DNA Gel Extraction Kit and subsequently sequenced by Eurofins Genomics Germany GmbH.

Phylogenetic trees were constructed for the CP of CMV, the RdRp of TAV and the polyprotein gene for PVCV. Viral sequences obtained in this study were aligned with reference sequences from NCBI using GENEIOUS software (v4.8.5, Dottmatics, Boston, MA, USA) (Table S4). Phylogenetic tree construction was performed using the Maximum Likelihood method and Tamura–Nei (1993) model [35] with 1000 bootstrap replicates for statistical support. Evolutionary analyses were conducted in MEGA12 utilizing up to 4 parallel computing threads [36,37].

## 5. Conclusions

Overall, our analysis revealed that viral distribution and diversity in petunia plants are not always associated with phenotypic expression. This requires integrated virome studies that take into account both the stage of individual development and the physiological state of the host, as well as environmental conditions, in order to fully understand the interactions between the plant and the virus and the dynamics of the diseases. On the other hand, our observations confirm the importance of metatranscriptome analysis for virus transmission in asymptomatic plants as well as in mixed infections. The created in vitro model system preserves the viruses detected in the original source, which allows their propagation and subsequent study.

## Figures and Tables

**Table 1:** Identification of plant viruses in individual petunias by PCR/RT-PCR.

	Market							In Vitro				
Sympt. plants	41 Ms	42 Ms	43 Ms	47 Ms	48 Ms	49 Ms	41 Is	42 Is	43 Is	47 Is	48 Is	49 Is
TAV	+	+	−	−	+	−	+	+	−	−	+	−
CMV	+	+	−	−	+	+	+	+	+	+	+	+
PVCV	+	+	−	+	+	+	+	+	+	+	+	+
	Market							In vitro				
Asympt. plants	31 Mas	33 Mas	35 Mas	40 Mas	51 Mas	53 Mas	31 Ias	33 Ias	35 Ias	40 Ias	51 Ias	53 Ias
TAV	−	+	+	−	−	−	−	+	+	−	−	−
CMV	+	+	+	+	+	+	+	+	+	+	+	+
PVCV	−	+	+	+	+	+	+	+	+	+	+	+

Mas = asymptomatic market; Ms = symptomatic market; Ias = asymptomatic in vitro; Is = symptomatic in vitro.

**Table 2:** Intersection of virus distribution between petunia sample groups. Shared virus distributions among different petunia sample groups were analyzed using UpSet plots to visualize the intersections; virus names are presented as listed in the Kraken database.

Intersection	Viruses
Ias & Is & Mas & Ms	cucumber mosaic vírus; tomato aspermy vírus; petunia vein clearing vírus
Mas & Ms	Petunia exserta mitovirus 1, * *Partitiviridae* sp.; cherry leaf roll virus
Is & Mas & Ms	* *Bacteriophage* sp.
Mas	apple mosaic virus; Botrytis cinerea alpha-like virus 1; Botrytis cinerea mitovirus 1; grapevine associated narnavirus-1
Ias	Erysiphe necator associated partitivirus 2; Erysiphales ourmia-like virus 2; Erysiphe necator ourmia-like virus 82; Erysiphe necator associated ourmia-like virus 82; * *Caudoviricetes* sp.

Mas = asymptomatic market; Ms = symptomatic market; Ias = asymptomatic in vitro; Is = symptomatic in vitro. * Viruses with not only plant hosts (*Partitiviridae* sp.) and bacteriophages are represented in the virome analyses software outputs as species in a certain genus or a family (*Bacteriophage* sp., *Caudoviricetes* sp., respectively) are listed in the table as in the original outputs.

## Data Availability

The raw HTS data generated in this study have been deposited in the NCBI Sequence Read Archive (SRA) under the BioProject accession number PRJNA1276299. The data are publicity available.

## Supplementary Materials

The following supporting information can be downloaded at: https://www.mdpi.com/article/10.3390/plants14162597/s1, Supplementary Table S1: Parameters of the rapid chlorophyll *a* fluorescence (OJIP test) and chlorophyll content (Chl) of asymptomatic market collected plants and symptomatic market petunia plants; Supplementary Table S2: Summary of sequencing results and classification by Pavian software (v1.0); Supplementary Table S3: Distribution of viral reads at family (a), genus (b), and species (c) ranks of *Petunia hybrida* samples belonging to Ia (in vitro asymptomatic); I (in vitro symptomatic); Ma (market asymptomatic); M (market, symptomatic) tested plants; Supplementary Table S4: List of viral isolates used for respective phylogenetic analyses with accession numbers, host species, and country of submission or origin for: (a) cucumber mosaic virus (b) tomato aspermy virus and (c) petunia vein clearing virus; Supplementary Table S5: Primers, used to amplify tomato aspermy virus, cucumber mosaic virus and petunia vein clearing virus in *P. hybrida* samples; Supplementary Table S6: RT-PCR, one and two step protocols used for the detection of cucumber mosaic virus, tomato aspermy virus and petunia vein clearing virus in *P. hybrida* samples.
