# Supplementary material for: Unveiling the Petunia hybrida Virome: Metatranscriptomic Profiling from the Bulgarian Market and In Vitro Cultures
Source: Plants (Basel). 2025 Aug 21;14(16):2597. doi: 10.3390/plants14162597 (PMC12389112; doi:10.3390/plants14162597)
Supplement: Supplementary file 1 [file plants-14-02597-s001.zip › plants-3794198-supplementary.pdf]

Supplementary data

Table S1. Parameters of the rapid chlorophyll *a* fluorescence (OJIP test) and chlorophyll content (Chl) of asymptomatic market collected plants and symptomatic market petunia plants.

Parameters	asymptomatic plants	symptomatic plants
F_0	241± 7 b	334±25 a
F_M	1398 ± 44 a	1397 ± 83 a
F_V	1157 ± 38 a	1063± 53 b
F_V/F_M	0.828 ± 0.006 a	0.761 ± 0.006 [illegible]
ψE_0	0.62 ± 0.04 a	0.50 ± 0.03 b
φE_0	0.51 ± 0.04 a	0.38 ± 0.04 b
δR_0	0.30 ±0.01 a	0.25 ± 0.02 b
PI abs	5.16 ± 0.87 a	2.56 ± 0.83 b
PI total	2.37 ± 0.15 a	0.85 ±0.12 b
Chl content	20.17 ± 0.96 a	7.91 ± 1.54 b

Means ± standard error (SE); means followed by the same letter are not significantly different at $p \le 0.05$ (n=5)

Definitions of the recorded and calculated chlorophyll *a* fluorescence parameters according to Strasser and Strasser [1995] and Goltsev et al. (2016):

F_0~ F20μs- Minimum fluorescence, when all PSII reaction centers (RCs) are open;

F_J- Fluorescence at the J-step (2 ms) of the O-J-I-P transient

F_I- Fluorescence at the I-step (30 ms) of the O-J-I-P transient

$F_M = F_P$ Maximum recorded fluorescence at the P-step when all RCs are closed

$V_J = (F_J – F_0)/(F_M – F_0)$ Relative variable fluorescence at the J-step

$F_V = F_M – F_0$ Variable fluorescence

$\psi_{E0} = 1 – V_J$ Probability (at t = 0) that a trapped exciton moves an electron into the electron transport chain beyond QA- $\varphi_{E0} = (1- F_J/F_M)$ Quantum yield (at t = 0) for electron transport from QA- to plastoquinone

$\delta R_0 = (1 - V_I)/(1 - V_J)$ Efficiency/probability (at t = 0) with which an electron from the intersystem carriers moves to reduce end electron acceptors at the PSI acceptor side

PI_{ABS} Performance index of PSII based on absorption

$PI_{total}=PI_{ABS} x\ \delta R_0/ (1 – \delta R_0)$ Performance index of electron flux to the final PSI electron acceptors, i.e., of both PSII and PSI

Table S2. Summary of sequencing results and classification by Pavian software (v1.0).

Sample	Number of raw reads	Classified reads	Unclassified reads	Viral reads
Ms (Market symptomatic petunias)	48,897,157	88.6%	11.4%	0.0402%
Mas (Market asymptomatic petunias)	47,025,103	82.9%	17.1%	0.00531%
Is (*In vitro* symptomatic petunias)	48,016,732	85.4%	14.6%	0.155%
Ias (*In vitro* asymptomatic petunias)	46,380,965	87.5%	12.5%	0.171%

Table S3. Distribution of viral reads at family (a), genus (b), and species (c) ranks of *Petunia hybrida* samples belonging to Ia (*in vitro* asymptomatic); I (*in vitro* symptomatic); Ma (market asymptomatic); M (market, symptomatic) tested plants.

(a)

			clade reads					clade reads %					
Name	Rank	TID	Max	Ia	I	Ma	M	Max	Ia	I	Ma	M	[illegible]
Bromoviridae	F	39740	22976	22976	19278	750	287	96.97	96.97%	30.54%	54.15%	40.42%	[illegible]
Mitoviridae	F	2732892	257	4	9	105	257	36.2	0.01688%	0.01426%	7.581%	36.2%	[illegible]
Caulimoviridae	F	186534	43821	418	43821	353	70	69.42	1.764%	69.42%	25.49%	9.859%	[illegible]
Partitiviridae	F	11012	42	23		30	42	5.915	0.09707%		2.166%	5.915%	[illegible]
Secoviridae	F	675072	32			17	32	4.507			1.227%	4.507%	[illegible]
Totiviridae	F	11006	17	1		17	9	1.268	0.00422%		1.227%	1.268%	[illegible]
Virgaviridae	F	675071	11	6	11	8	4	0.5776	0.02532%	0.01742%	0.5776%	0.5634%	[illegible]
Betaflexiviridae	F	675068	3				3	0.4225				0.4225%	[illegible]
Botourmiaviridae	F	2560063	253	253	3	13	2	1.068	1.068%	0.004752%	0.9386%	0.2817%	[illegible]
Mimiviridae	F	549779	2			1	2	0.2817			0.0722%	0.2817%	[illegible]
Narnaviridae	F	186766	43	6		43	1	3.105	0.02532%		3.105%	0.1408%	[illegible]
Polydnaviriformidae	F	2946196	1		1		1	0.1408		0.001584%		0.1408%	[illegible]
Kitaviridae	F	2560061	3	3	1			0.01266	0.01266%	0.001584%			[illegible]
Tombusviridae	F	39738	9	1		9		0.6498	0.00422%		0.6498%		[illegible]
Mesoniviridae	F	1312872	1	1				0.00422	0.00422%				[illegible]

(b)

Name	Rank	TID	clade reads Max	clade reads Ia	clade reads I	clade reads Ma	clade reads M	clade reads % Max	clade reads % Ia	clade reads % I	clade reads % Ma	clade reads % M	[illegible]
Petuvirus	G	186845	43821	418	43821	353	70	69.42	1.777%	69.42%	26.54%	10.42%	[illegible]
Cucumovirus	G	12304	22975	22975	19278	734	279	97.7	97.7%	30.54%	55.19%	41.52%	[illegible]
Duamitovirus	G	2948686	257	3	6	83	257	38.24	0.01276%	0.009505%	6.241%	38.24%	[illegible]
Ourmiavirus	G	186783	96	96	2	7	1	0.5263	0.4082%	0.003168%	0.5263%	0.1488%	[illegible]
Narnavirus	G	186767	43			43	1	3.233			3.233%	0.1488%	[illegible]
Nepovirus	G	12270	32			17	32	4.762			1.278%	4.762%	[illegible]
Totivirus	G	11007	16	1		16	9	1.339	0.004252%		1.203%	1.339%	[illegible]
Ilarvirus	G	12316	16			16	7	1.203			1.203%	1.042%	[illegible]
Pahexavirus	G	1982251	14	14	2	3	3	0.4464	0.05953%	0.003168%	0.2256%	0.4464%	[illegible]
Mitovirus	G	186768	12			12		0.9023			0.9023%		[illegible]
Tobamovirus	G	12234	11	6	11	7	4	0.5952	0.02551%	0.01743%	0.5263%	0.5952%	[illegible]
Alphavirus	G	11019	11			11		0.8271			0.8271%		[illegible]
Umbravirus	G	39734	7			7		0.5263			0.5263%		[illegible]
Unuamitovirus	G	2948940	5			5		0.3759			0.3759%		[illegible]
Barnavirus	G	39742	3			3		0.2256			0.2256%		[illegible]

(c)

Name	Rank	TID	clade reads Max	clade reads Ia	clade reads I	clade reads Ma	clade reads M	clade reads % Max	clade reads % Ia	clade reads % I	clade reads % Ma	clade reads % M	[illegible]
Petuvirus venapetuniae	S	3048265	43821	418	43821	353	70	70.05	1.826%	70.05%	26.8%	10.04%	[illegible]
Cucumber mosaic virus	S	12305	21410	21410	18490	31	11	93.51	93.51%	29.56%	2.354%	1.578%	[illegible]
Tomato aspermy virus	S	12315	727	727	164	599	216	45.48	3.175%	0.2622%	45.48%	30.99%	[illegible]
Duamitovirus peex1	S	2955799	257	3	6	51	257	36.87	0.0131%	0.009591%	3.872%	36.87%	[illegible]
Erysiphales ourmia-like virus 2	S	2719868	108	108	1			0.4717	0.4717%	0.001599%			[illegible]
Caudoviricetes sp.	S	2832643	81	81	6	9	4	0.6834	0.3538%	0.009591%	0.6834%	0.5739%	[illegible]
Erysiphe necator ourmia-like virus 82	S	2950842	40	40	1			0.1747	0.1747%	0.001599%			[illegible]
Grapevine associated narnavirus-1	S	908834	38			38		2.885			2.885%		[illegible]
Partitiviridae sp.	S	1955170	38			28	38	5.452			2.126%	5.452%	[illegible]
Erysiphe necator associated ourmia-like virus 82	S	2741862	32	32				0.1398	0.1398%				[illegible]
Nepovirus avii	S	3047741	32			17	32	4.591			1.291%	4.591%	[illegible]
Erysiphe necator associated partitivirus 2	S	2737084	23	23				0.1004	0.1004%				[illegible]
Duamitovirus boci1	S	2955769	18			18		1.367			1.367%		[illegible]
Bacteriophage sp.	S	38018	17	5	16	17	13	1.865	0.02184%	0.02558%	1.291%	1.865%	[illegible]
Ilarvirus ApMV	S	12319	16			16	7	1.215			1.215%	1.004%	[illegible]

Table S4 List of viral isolates used for respective phylogenetic analyses with accession numbers, host species, and country of submission or origin for: (a) cucumber mosaic virus (b) tomato aspermy virus and (c) petunia vein clearing virus.

(a) CMV accessions

№	name	country of submission or origin*	host/isolation source
1	D10538.1	USA	*Nicotiana tabacum* cv Xanthi
2	LN810059.1	Greece	*Citrus lanatus*
3	JF918964.1	USA	*Vinca minor*
4	KU695261.1	Iran	*Dimorphotheca caulescens*
5	JF918966.1	USA	*Vinca minor*
6	AJ131624.1	The Netherlands	bean
7	PV037670.1	Bulgaria	*Petunia hybrida*
8	AY374327.1	Brazil	*Impatiens walleriana*
9	JF918967.1	USA	*Vinca minor*
10	AY380532.1	Brazil	*Salvia splendens*
11	AY377584.1	Brazil	*Hypocyrta nervosa*
12	AY376840.1	Brazil	*Catharanthus roseus*
13	AY380533.1	Brazil	chrysanthemum
14	AJ131623.1	The Netherlands	*Gladiolus* cv Peter Pears
15	AY374328.1	Brazil	*Lilium* sp.
16	AJ131616.1	The Netherlands	lily asiatic hybrid cv Connecticut King
17	AJ131619.1	The Netherlands	lily in Taiwan
18	AJ131615.1	The Netherlands	lily asiatic hybrid cv Polyanna
19	AJ131618.1	The Netherlands	lily cv New Butterfly in Taiwan
20	AJ131617.1	The Netherlands	lily asiatic hybrid cv Sun Ray
21	LN810058.1	Greece	*Citrullus lanatus*
22	LN810060.1	Greece	*Citrullus lanatus*
23	U20219.1	USA	*Lycopersicon esculentum*
24	AJ131625.1	The Netherlands	amaranthus in Taiwan
25	AJ131626.1	The Netherlands	larkspur in Taiwan
26	AJ131627.1	The Netherlands	lisianthus in Taiwan

27	AJ810262.1	Germany	bean from Bulgaria
28	AM396983.1	India	*Tagetes erecta*
29	JF918965.1	USA	*Vinca minor*
30	AJ131620.1	The Netherlands	ornamental plant
31	M21464.1	Australia	*Capsicum annuum*
32	AJ131622.1	The Netherlands	alstroemeria
33	AJ131621.1	The Netherlands	ornamental plant

(b) TAV accessions

№	name	country of submission or origin*	host/isolation source
1	LC380670.1	Japan	*Solanum lycopersicum*
2	LC380673.1	Japan	*Chrysanthemum* sp.
3	HQ424164.1	China	*Nicotiana benthamiana*
4	AJ320274.1	South Korea	Crysanthemum
5	LC634032.1	Japan	*Chrysanthemum morifolium*
6	MN841292.1	China	*Chrysanthemum indicum*
7	LC380676.1	Japan	*Solanum lycopersicum*
8	PV037669.1	Bulgaria	*Petunia hybrida*
9	NC003838.1	Spain	*Chrysanthemum* sp
10	OK558776.1	Canada	CPVC-286-1
11	OL311686.1	Germany	chrysanthemum
12	MW582783.1	Germany	*Solanum lycopersicum*
13	OK558777.1	Canada	CPVC-288-1
14	OP924361.1	Russia	*Chrysanthemum morifolium*
15	KT757537.1	Iran	petunia
16	MW080948.1	Germany	*Solanum lycopersicum*
17	OP924358.1	Russia	*Chrysanthemum morifolium*
18	OL472070.1	Slovenia	*Solanum lycopersicum*
19	KF432414.1	China	*Chrysanthemum sp.*
20	MW080947.1	Germany	*Solanum lycopersicum*
21	MW080949.1	Bulgaria	*Solanum lycopersicum*

(c) PVCV accessions

№	name	country of submission or origin*	host/isolation source
1	PQ787219.1	Bulgaria	*Petunia hybrida*
2	AY228106.1	The Netherlands	*Petunia hybrida* cv W138
3	AY333912.1	The Netherlands	*Petunia hybrida*
4	PV599763.1	Bulgaria	*Petunia hybrida*
5	MN399814.1	South Korea	*Petunia hybrida*
6	MK472692.1	South Korea	*Petunia hybrida*
7	NC001839.2	USA	*Petunia hybrida* cv Himmelsroeschen

The host names are presented as they appear in the NCBI database with a correction for cultivar abbreviation according to the proper botanical nomenclature. *The "Country" column indicates the country where the sequence was reported, which may differ from the actual geographic origin of the virus isolate.

Table S5. Primers, used to amplify cucumber mosaic virus (CMV, *Cucumovirus CMV)*, tomato aspermy virus (TAV, *Cucumovirus TAV)* and petunia vein clearing virus (PVCV, *Petuvirus venapetuniae)* in *P. hybrida* samples.

Primer	Sequence 5′ – 3′	Amplicon size	Tm (°C)	reference
TAV-F	CGCTGTTTCAACGCTTTCAACG	852bp	53°C	Chen *et al.*2015
TAV-R	CCACGACCAACAATCAAATCAC			
CMV-F	GCCACCAAAAATAGACCG	593bp	54°C	Chen *et al*, 2015
CMV-R	ATCTGCTGGCGTGGATTTCT			
PVCV-F	GAGGTCAGAGCAAGTCAGAGG	736 bp	60°C	Gera *et al*, 2000
PVCV-R	GTAATGATTTGACTTGTTGAG			

Zhao, X., Liu, X., Ge, B. et al. A multiplex RT-PCR for simultaneous detection and identification of five viruses and two viroids infecting chrysanthemum. Arch Virol 160, 1145–1152 (2015). https://doi.org/10.1007/s00705-015-2360-z
A. Gera, N. Sikron, J. Cohen, and M. Zeidan First Report of *Petunia vein clearing virus* in Israel Plant Disease 2000 84:2, 201-201 https://doi.org/10.1094/PDIS.2000.84.2.201B

Table S6. RT-PCR, one and two step protocols used for the detection of cucumber mosaic virus, tomato aspermy virus and petunia vein clearing virus in *P.hybrida* samples.

One step RT-PCR<br>for virus identification		Two step RT-PCR<br>for Sanger sequencing	
cDNA synthesis	70°C/5 min<br>24°C/5 min<br>50°C/60 min	cDNA synthesis<br>(SCRIPT Reverse transcriptase)	70°C/5 min<br>4°C/5 min<br>42°C/10min<br>50°C/60 min<br>70°C/5 min
initial denaturation	95°C/5 min	initial denaturation	95°C/2 min
denaturation	95°C/10 s	denaturation	95°C/20 s
annealing	53°-60°C/20 s	annealing	53°-60°C/20 s
elongation	72°C/60 s	elongation (Pfu pol)	68°C/60 s
final elongation	72°C/5 min	final elongation	68°C/2 min
